# Complete Genome Sequence of *Salmonella enterica* subsp. *enterica* Serovar Enteritidis Strain SEJ

**DOI:** 10.1128/genomeA.01084-14

**Published:** 2014-10-23

**Authors:** K. A. Bishop-Lilly, K. G. Frey, H. E. Daligault, K. W. Davenport, D. C. Bruce, P. S. Chain, S. R. Coyne, O. Chertkov, T. Freitas, J. Jaissle, G. I. Koroleva, J. T. Ladner, T. D. Minogue, G. F. Palacios, C. L. Redden, Y. Xu, S. L. Johnson

**Affiliations:** a Naval Medical Research Center (NMRC)–Frederick, Fort Detrick, Maryland, USA; b Henry M. Jackson Foundation, Bethesda, Maryland, USA; c Los Alamos National Laboratory (LANL), Los Alamos, New Mexico, USA; d Diagnostic Systems Division (DSD), United States Army Medical Research Institute of Infectious Diseases (USAMRIID), Fort Detrick, Maryland, USA; e Center for Genome Sciences (CGS), USAMRIID, Fort Detrick, Maryland, USA

## Abstract

*Salmonella enterica* constitutes a group of enteric pathogens with a broad host range, including humans, reptiles, and birds. *S. enterica* subsp. *enterica* is a common cause of inflammatory diarrhea in humans. We present the draft genome of *S. enterica* subsp. *enterica* serovar Enteritidis strain SEJ, including a 59-kbp plasmid.

## GENOME ANNOUNCEMENT

*Salmonella enterica* constitutes a group of enteric pathogens with a broad host range, including humans, reptiles, and birds. Of this group, *S. enterica* subsp. *enterica* is the predominant pathogen of birds and mammals. Disease manifestations and/or severity can vary by host status and age but a typical manifestation would be inflammatory diarrhea (reviewed in reference 1). Tracking enteric pathogens such as these for epidemiological purposes has traditionally been performed via pulsed-field gel electrophoresis (PFGE); however, for the serovar Enteriditis, phylogenetic analysis based on whole-genome sequence data was recently shown to provide increased resolution (2). Accordingly, there is utility in increasing the number of high-quality references available. We present here the draft genome of *S. enterica* subsp. *Enterica* serovar Enteritidis strain SEJ, sequenced to 314× total draft coverage.

A QIAgen Genome Tip-500 was used to extract high-quality genomic DNA from a purified isolate. Specifically, nucleic acid was extracted from a 100-mL bacterial culture at stationary phase. This draft genome was derived via a combination of Illumina library technologies (1). A 100-bp unpaired library was constructed (312-fold genome coverage) as well as a long-insert paired-end library (7,944 ± 2,066-bp insert and 2-fold genome coverage). The paired-end data were assembled in Newbler (3) and those consensus sequences computationally shredded into 2 kbp overlapping fake reads (shreds). The standard Illumina sequencing data were assembled with VELVET (2) and the consensus sequence computationally shredded into 1.5-kbp overlapping shreds. All data were additionally assembled together in Allpaths (4) and the consensus sequences computationally shredded into 5-kbp overlapping shreds. All of the shreds were integrated using parallel phrap (High Performance Software, LLC). Possible misassemblies were corrected and repeat regions verified using in-house scripts and manual editing in Consed (3–6).

By these methods the chromosome and plasmid sequence for this strain were assembled into a single contig each. Annotation was performed using an Ergatis-based (7) workflow with minor manual curation. The *S. enterica* SEJ chromosome (CP008928) is 4,678,927 bp (52.2% G+C content) and encodes 4,341 coding sequences, 21 rRNAs, and 82 tRNAs. The 59,372-bp plasmid (CP008927) has 52.0% G+C content and encodes 77 CDSs. This draft genome expands the repertoire of *Salmonella* whole-genome sequences available for comparative analysis.

### Nucleotide sequence accession numbers.

The NCBI accession no. for the *S. enterica* SEJ chromosome is CP008928 and for the plasmid is CP008927.
